# Data sharing from pharmaceutical industry sponsored clinical studies: audit of data availability

**DOI:** 10.1186/s12916-018-1154-z

**Published:** 2018-09-28

**Authors:** Ashley M. Hopkins, Andrew Rowland, Michael J. Sorich

**Affiliations:** 1 Flinders Centre for Innovation in Cancer, College of Medicine and Public Health, Flinders University, Flinders Drive, Bedford Park, Adelaide, South Australia 5042 Australia; 2 Department of Clinical Pharmacology, College of Medicine and Public Health, Flinders University, Flinders Drive, Bedford Park, Adelaide, South Australia 5042 Australia

**Keywords:** Data sharing, Clinical trials, Pharmaceutical industry

## Abstract

**Background:**

Clinical trial transparency is important to participants, trialists, publishers, and regulators, and there have been recent major policy changes by the pharmaceutical industry regarding clinical study data sharing. However, it is unknown if these changes are enabling independent researchers to access participant-level data from prominent contemporary clinical trials sponsored by the pharmaceutical industry 2 years after publication of the primary results.

**Main text:**

PubMed and ClinicalTrials.gov were searched to identify clinical trials of medicines sponsored by the pharmaceutical industry and first published between 1 July 2015 and 31 December 2015 in the top 10 general and internal medical journals by impact factor. For each clinical trial, the eligibility of independent researchers to request participant-level data was identified via the sponsor having a data sharing policy/process and a positive response to an enquiry.

Fifty-six publications reporting on 61 industry-sponsored clinical trials were identified, of which 32 (52%) had a public data sharing policy/process and 9 (15%) were confirmed eligible for data sharing. Industry sponsors within the top 25 by global sales were more likely to have a data sharing policy (93% vs 10%), and there was a trend towards increased data sharing eligibility (23% vs 4%). Twenty-six studies were explicitly confirmed as ineligible for data sharing. The two most common data sharing policy conditions that prevented sharing of data for published results were the exclusion of studies that had ongoing follow-up of the published results and the exclusion of studies of medicines that have not yet achieved regulatory approval in the USA and the European Union.

**Conclusions:**

Fifteen percent of the sampled clinical trials were available for data sharing 2 years after publication of primary results of the trial. Key issues limiting data sharing include a large proportion of industry sponsors who do not have a data sharing policy/process, and data sharing policy conditions that exclude access on the basis of ongoing follow-up and regulatory activity.

**Electronic supplementary material:**

The online version of this article (10.1186/s12916-018-1154-z) contains supplementary material, which is available to authorized users.

## Background

Clinical trial transparency is important to participants, sponsors, trialists, publishers, and regulators [1–10]. Responsible sharing of participant-level data (also known as individual participant data, IPD) is one aspect of clinical trial transparency which enables novel secondary analyses, verification of results, and optimisation of future study designs [1, 11, 12]. Effective 1 January 2014, the biopharmaceutical industry — via the Pharmaceutical Research and Manufacturers of America (PhRMA) and the European Federation of Pharmaceutical Industries and Associations (EFPIA) — endorsed a commitment to share de-identified IPD for approved medicines and indications upon request with qualified researchers [3].

This endorsement was an important milestone in the pharmaceutical industry transitioning towards data sharing. It has sparked significant discussion and debate on several issues related to data sharing, including balancing the ease of access to shared data and the need to safeguard participant anonymity [13–16], ensuring participants continue to enrol in clinical trials [10], developing the required resources and support systems to facilitate valid research output [15–18], the opportunity cost of data sharing [19, 20], and mechanisms to provide adequate attribution to original investigators [16, 20, 21]. However, little is known on the eligibility of independent researchers to request access to participant-level data. Evidence to date suggests that the scientific review process of submitted proposals to access clinical trial data is not a barrier [19, 22], and thus a key step is whether a study is in scope. Whether a study is in scope for data sharing depends on whether the trial sponsor has a data sharing policy and, if so, the specific conditions of the policy. A recent audit of 42 selected industry sponsors found that 74% had a policy to share IPD, but it did not evaluate the conditions or the degree to which the policies affected published trials from being in scope to share [23]. Herein the aim was to evaluate the proportion of prominent contemporary clinical trials sponsored by the pharmaceutical industry available for sharing of participant-level data with independent researchers 2 years after first publication of the primary results.

## Main text

### Audit methods

A structured search of PubMed was undertaken on 9 August 2017 to identify primary publications for industry-sponsored clinical trials investigating medicines registered on ClinicalTrials.gov and first published (including electronic publishing date) between 1 July 2015 and 31 December 2015 in the top 10 general and internal medical journals by impact factor [24]. The primary sponsor, primary endpoint, primary completion date, and final completion date for the identified clinical trials were collated from ClinicalTrials.gov. The websites FDA.gov and EMA.europa.eu were searched on 1 October 2017 to identify if the medicine(s) being investigated in each of the trials were FDA or European Medicines Agency (EMA) registered. On 18 December 2017, PMLiVE.com was searched to identify sponsors within or below the top 25 pharmaceutical companies by global sales [25]. PhRMA.org and EFPIA.eu were also searched on 18 December 2017 to identify PhRMA and/or EFPIA member sponsors.

Between 9 August 2017 and 23 August 2017, the website of each clinical trial sponsor was searched to identify any public data sharing policy or process. Eligibility of a trial for data sharing was defined as being in scope with respect to the sponsor’s data sharing policy. Eligibility for data sharing was confirmed by either public listing of the study as available for data sharing or a positive response to an enquiry directed to the trial sponsor in conjunction with a data sharing policy. If the trial was not eligible for data sharing, we requested details of the reason and, if appropriate, when the trial would become eligible. Replies within a pre-specified 3-month period from the initial enquiry were included in the main analysis (see Additional file 1: Supplementary Methods).

Planned exploratory analyses evaluated trial data sharing eligibility stratified by sponsoring pharmaceutical company size (within and below the top 25 by global sales [25]), sponsoring pharmaceutical company PhRMA/EFPIA membership (members and non-members), and therapeutic area of the pharmaceutical intervention.

### Audited publications, trials, and sponsors

Fifty-six publications met the inclusion criteria, of which 36 were published in the *New England Journal of Medicine*, 13 in *The Lancet*, 5 in the *Journal of the American Medical Association*, and 2 in the Annals of Internal Medicine. The 56 publications reported 61 clinical trials, of which 35 were sponsored by a pharmaceutical company within the top 25 by global sales and 26 by a pharmaceutical company below the top 25 by global sales [25]. Thirty clinical trials were sponsored by a PhRMA/EFPIA member company and 31 by a non-PhRMA/EFPIA member company. Forty-eight (79%) of the clinical trials investigated a medicine and indication that was approved by either/both the FDA or/and the EMA as of 1 October 2017.

### IPD availability

**Fig. 1:**
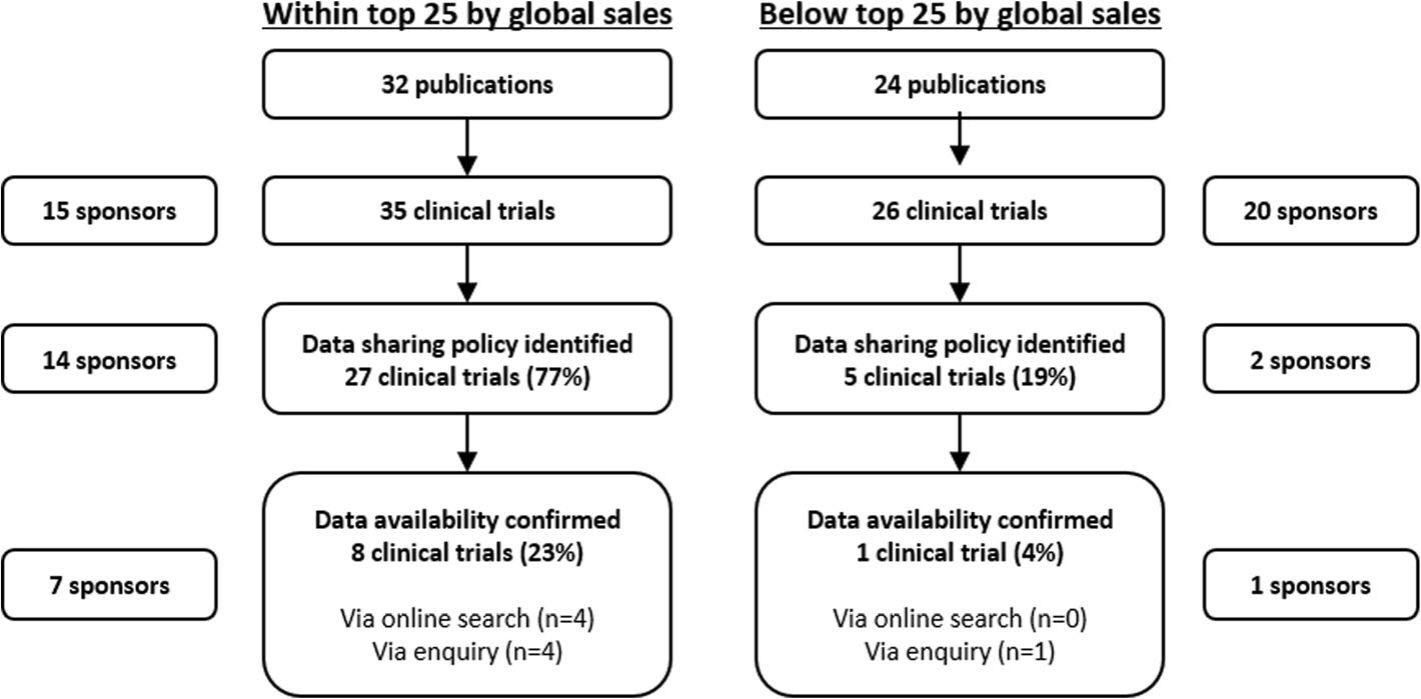
Eligibility for data sharing by global sales of sponsoring pharmaceutical company

Of the 61 industry-sponsored clinical trials to which the eligibility of independent researchers to request participant-level data was investigated, a public policy for data sharing was identified for 32 trials (52%), and 9 trials (15%) were confirmed as being eligible for data sharing (Additional file 1: Supplementary Results). Industry sponsors within the top 25 by global sales were more likely to have a publicly available data sharing policy (93% vs 10%) than those below the top 25 by global sales, and there was a trend towards increased data sharing eligibility (23% vs 4%) (Fig. 1). Industry sponsors that were members of PhRMA/EFPIA were significantly more likely to have a data sharing policy (94% vs 5%) and confirm trial data sharing eligibility (30% vs 0%) than non-members (Additional file 1: Figure S1 and Table S2).

For 26 clinical trials (43%) no confirmation of data sharing eligibility was forthcoming, including 4 trials for which eligibility enquiries were not permitted without a full study proposal, 10 trials for which no response was acquired within 3 months to a specified data sharing enquiry process, and 12 trials for which no response was acquired within 3 months to a generic sponsor contact (Table 1). Data sharing was confirmed as not available for 26 trials (43%) (Table 1). The most common reason was that the sponsor does not share IPD, and the most common conditional reasons were that the study is still ongoing (i.e. follow-up ongoing after publication of a primary result) and that the medicine is not approved by both the EMA and FDA (Table 1). Two clinical trials were confirmed eligible for data sharing after the 3-month enquiry period had expired (confirmed 5.3 and 6.6 months after initial enquiry). One clinical trial was confirmed as ineligible for data sharing after the 3-month enquiry period had expired (confirmed 5 months after initial enquiry) on the basis that the trial was still ongoing. No additional trials were confirmed as eligible/ineligible for data sharing at 9 months after initial enquiry.

**Table 1:** Breakdown of clinical trials for which eligibility for data sharing was not confirmed by global sales of sponsoring pharmaceutical company

Reason	Within top 25 by global sales (*n* = 35)	Below top 25 by global sales (*n* = 26)
Confirmed data sharing not available	17 (49%)	9 (35%)
• Sponsor does not share IPD	8 (23%)	7 (27%)
• Study is still ongoing	5 (14%)	0
• Medicine not approved by both EMA and FDA, or ongoing regulatory submission	4 (11%)	1 (4%)
• Medicine no longer in development	0	1 (4%)
Unable to confirm eligibility/ineligibility	10 (29%)	16 (62%)
• No response within 3 months to specified data sharing enquiry process^a,b^	6 (17%)	4 (15%)
• No response within 3 months to generic sponsor contact^c^	0	12 (46%)
• Full research proposal required to assess eligibility	4 (11%)	0

Data specified as number of trials (% of the total number of trials assessed by global sales status)

^a^One clinical trial (conducted by a company below the top 25 by global sales) was confirmed as ineligible for data sharing after the 3-month enquiry period had expired (confirmed 5 months after initial enquiry) on the basis that the trial was still ongoing

^b^Two clinical trials (one conducted by a company within, and one conducted by a company below the top 25 by global sales) were confirmed eligible for data sharing after the 3-month enquiry period had expired (confirmed 5.3 and 6.6 months after initial enquiry)

^c^No specific data sharing enquiry contact/process identified

Exploratory analysis indicated that 21 (34%) and 13 (21%) of the 61 clinical trials had not passed the trial completion date at the time of publication and 2 years after publication, respectively, with the highest frequency for oncology trials (85% and 62%, respectively) (Additional file 1: Table S3).

### Data sharing process

The data sharing process, including the enquiry process, was managed directly by the sponsor for 12 trials (7 sponsors), by ClinicalStudyDataRequest.com (CSDR) for 16 trials (8 sponsors), and by the Yale University Open Data Access (YODA) project for 4 trials (1 sponsor). For all 9 clinical trials eligible for data sharing, the data custodian was the sponsor listed on ClinicalTrials.gov. For the 39 trials (64%) for which eligibility or ineligibility for data sharing was confirmed, the median (range) response time was 5 days (0–49 days).

## Discussion

To our knowledge, this is the largest structured assessment of the eligibility of independent researchers to request IPD from a broad selection of contemporary clinical trials that have been recently published. This study was also the first to identify data sharing policy decisions that have a negative impact on trial data sharing and to evaluate and identify the influence of PhRMA/EFPIA membership of industry sponsors on data sharing public policy and trial eligibility for data sharing.

With respect to study limitations, the eligibility of data sharing was investigated for clinical trials published 2–2.5 years ago in the top 10 general and internal medicine journals by impact factor. This time period was chosen as a reasonable balance between opportunity for trialists to publish key findings and independent data analysts to access data to contemporary trials which are affecting current health care — noting that 48 (79%) of the 61 clinical trials investigated a medicine and indication that was approved by either/both the FDA or/and EMA as of 1 October 2017. Eligibility may be different at other time points following publication, which is relevant as the Institute of Medicine committee recommend IPD sharing no later than 6 months after publication [26], and the biopharmaceutical industry statement on principles for data sharing specifies no timeframe [3]. Eligibility may also differ for clinical trials not published in the sampled journals or for the portion of clinical trials that go unpublished. Additionally, all 9 clinical trials that were identified as eligible for data sharing required a review board to accept a submitted research proposal prior to granting access to IPD, and this process was not assessed. Future studies should investigate the time from proposal submission to data sharing, data completeness upon sharing, researcher support initiatives, and data sharing eligibility of non-industry-sponsored trials. The importance of such investigations was recently reinforced by Naudet et al. [27], who conducted a primary outcome reanalysis study in which only 46% (17 of 37) of trial authors provided complete IPD to randomised controls trials published in *The BMJ* and *PLOS Medicine*, journals with a strong data sharing policy (note that 26 of 37 sampled trials had no industry funding) [28].

A recent audit of 42 selected industry sponsors reported that 74% had a policy for clinical trial data sharing [23]. However, there was no assessment of how often clinical trials would be eligible for data sharing under reasonable circumstances (e.g. 2 years after publication). In comparison, 52% of our sampled industry sponsors had a public data sharing policy. The difference was largely driven by 46% of our sampled sponsors being a PhRMA or EFPIA member, compared with 81% in the prior study [23]. Thus, while the PhRMA and EFPIA jointly committed to sharing de-identified IPD for approved medicines on 1 January 2014 [3], this does not currently represent an industry-wide commitment, as the actioning of these principles for data sharing has been minimal by PhRMA and EFPIA non-members. Although PhRMA and EFPIA non-members are often smaller pharmaceutical companies, this study highlights the fact that they conduct and publish a significant portion (51% of trials in this sample) of contemporary clinical trials of medicines.

In 2015 Murugiah et al. assessed the data sharing eligibility of 60 clinical trials investigating cardiovascular medicines (> 5000 participants) sponsored by the top 20 revenue pharmaceutical companies [29]. The completion date of the trials sampled in the Murugiah et al. study ranged from 1 to 14 years prior to their enquiry submission [29]. Despite the current study sampling a wider range of medicines and sponsors and the focus on clinical trials published 2–2.5 years previous (avoiding historical data), our finding of 15% (9 of 61) of trials being eligible for data sharing was similar to the 15% (9 of 60) eligibility reported by Murugiah et al. This suggests that there has been minimal improvement in accessing industry-sponsored trial data in the 2.5 years since the Murugiah et al. study was conducted [29].

The two most common reasons for confirmed data sharing ineligibility according to a sponsor’s policy were that the study was ongoing and regulatory reasons. Many modern trials continue to follow up participants well after the primary outcome of the trial has been completed and the results published. For instance, medicines for advanced cancers commonly evaluate progression-free survival as the primary outcome but continue to follow up for overall survival. Registration of a medicine is usually based on the primary outcome, and hence widespread use of a drug may occur well before study follow-up is completed. This is problematic for many data sharing policies that indicate the trial is eligible for data sharing after (often 1 or 2 years after) a study is completed. Of the 61 clinical trials evaluated, a substantial proportion, particularly for oncology trials, had not passed the final completion date at publication and 2 years after publication, with completion dates ranging up to December 2020. If a study has results worthy of being published or has contributed to the registration of a medicine, it is unclear why incomplete follow-up for a secondary outcome should prevent sharing of the data supporting the published primary outcomes.

Additionally, five trials were confirmed as ineligible for data sharing because either the medicine was not approved by both the FDA and EMA or there was an ongoing regulatory submission. Registration by both the FDA and the EMA is a condition currently stipulated in data sharing policies of many industry sponsors [23, 26]. Arguably, once a medicine has been registered and is in widespread use in one jurisdiction, data sharing of the pivotal trials supporting the registration decision is in the public interest irrespective of the registration status in another jurisdiction.

Submission of a research proposal was required to assess eligibility of four clinical trials, and this was outside the scope of this study. Writing a research proposal is a major endeavour, and it is useful to be able to clarify whether a study is in scope for data sharing prior to developing a research proposal. As such, mandating a research proposal to assess data sharing eligibility for a specific clinical trial may discourage applications for data sharing.

Notably, of the 61 clinical trials to which eligibility for data sharing was enquired, no response/confirmation was received within 3 months for 22 trials, including 10 for which an explicit data sharing process was identified. This subgroup may partly represent trials in scope for data sharing; however, at 9 months after initial enquiry only 3 additional responses (2 trials eligible and 1 trial ineligible) were received, and thus the subgroup for the majority represents trials either ineligible for data sharing or trials that, due to severe process issues, were equated as ineligible for data sharing with independent researchers. As data sharing becomes more prevalent, this highlights the importance of considering and addressing the resource challenges faced by data custodians inherent in managing enquiries and proposals, preparing data for sharing, and appropriately managing data access [6, 9]. Data sharing systems and processes are not inexpensive to establish, and to date the cost has largely been absorbed by the pharmaceutical industry [19, 20].

## Conclusions

Based on a sample of 61 clinical trials of medicines sponsored by the pharmaceutical industry with primary results published between 1 July 2015 and 31 December 2015, 15% were confirmed as being eligible for data sharing with independent researchers 2–2.5 years after publication. A data sharing policy was identified for 52% of clinical trials. Notably, both trial data sharing eligibility and a public data sharing policy were much more common for industry sponsors that were members of PhRMA or EFPIA. Data sharing policy decisions of industry sponsors were identified that limit data sharing eligibility of trials. Notably, many data sharing policies limit data sharing for trials that undertake longer-term follow-up after completion and publication of the primary outcome. Additionally, many data sharing policies limit data sharing based on regulatory approval status of the medicine, often requiring approval by both the FDA and EMA.

## Additional files

Additional file 1: Supplementary methods and results. (DOCX 108 kb)

Additional file 2: Anonymised raw data. (XLSX 14 kb)

## Abbreviations

CSDR: ClinicalStudyDataRequest.com
EFPIA: European Federation of Pharmaceutical Industries and Associations
EMA: European Medicines Agency
FDA: US Food and Drug Administration
IPD: Individual participant data
PhRMA: Pharmaceutical Research and Manufacturers of America
YODA: Yale University Open Data Access
