# Supplementary material for: Data sharing from pharmaceutical industry sponsored clinical studies: audit of data availability
Source: BMC Med. 2018 Sep 28;16:165. doi: 10.1186/s12916-018-1154-z (PMC6161442; doi:10.1186/s12916-018-1154-z)
Supplement: Supplementary file 1 — Supplementary methods and results. (DOCX 108 kb) [file 12916_2018_1154_MOESM1_ESM.docx]

Additional File 1 for “Data Sharing from Clinical Studies: Assessment of Data Availability”

# Supplementary Methods

A structured search of PubMed was undertaken on 9 Aug 2017 to identify primary publications for industry sponsored clinical trials investigating medicines registered on ClinicalTrials.gov and first published (including electronic publishing date) between 1 July 2015 and 31 Dec 2015 in the top ten general and internal medical journals by impact factor - N Engl J Med, Lancet, JAMA, BMJ, Ann Intern Med, JAMA Intern Med, PLoS Med, J Cachexia Sarcopenia Muscle, BMC Med, or J Intern Med [1]. The primary sponsor, primary endpoint, primary completion date, and final completion date for the identified clinical trials were collated from ClinicalTrials.gov. FDA.gov and EMA.europa.eu were searched on 1 Oct 2017 to identify if the medicine(s) being investigated in each of the trials were FDA or EMA registered. On 18 Dec 2017, PMLiVE.com was searched to identify sponsors within or below the top 25 pharmaceutical companies by global sales [2]. PhRMA.org and EFPIA.eu were also searched on 18 Dec 2017 to identify PhRMA and/or EFPIA member sponsors.

Between 9 Aug 2017 and 23 Aug 2017, the website of each clinical trial sponsor was searched to identify any public data sharing policy or process. Eligibility of a trial for data sharing was defined as being in scope with respect to the sponsor’s data sharing policy. Eligibility for data sharing was confirmed by either public listing of the study as available for data sharing, or a positive response to an enquiry directed to the trial sponsor in conjunction with a data sharing policy. If the trial was not eligible for data sharing, details of the reason were requested and if appropriate when the trial would become eligible. For sponsors with a publically defined data sharing process the stated eligibility enquiry process was utilized. For trials where neither availability nor an enquiry process could be identified, the sponsoring company was first contacted to determine the most appropriate email to enquire regarding data sharing eligibility. If there was no response after two weeks, the general information email for the sponsor was used for enquiry. Replies within a pre-specified 3 month period from the initial enquiry were included in the main analysis.

Planned exploratory analyses evaluated trial data sharing eligibility stratified by sponsoring pharmaceutical company size (within and below the top 25 by global sales [25]), sponsoring pharmaceutical company PhRMA/EFPIA membership (members and non-members), and therapeutic area of the pharmaceutical intervention.

# Supplementary Results

Table S1: Breakdown of clinical trials for which eligibility for data sharing was confirmed

| **Therapeutic area** | **Sampled** | **Confirmed eligible for data sharing** |
| --- | --- | --- |
| Cardiovascular disease | 7 | 1 (14%) |
| Dermatology | 3 | 0 |
| Endocrine | 7 | 2 (29%) |
| Haematology | 7 | 1 (14%) |
| Infectious disease | 14 | 2 (14%) |
| Neurology | 1 | 1 (100%) |
| Oncology | 13 | 0 |
| Ophthalmology | 2 | 0 |
| Rheumatology | 7 | 2 (29%) |
| **Total** | **61** | **9 (15%)** |
| Data specified as: number of trials by therapeutic area (% of the number of trials assessed by therapeutic area) | | |

Figure S1: Eligibility for data sharing for PhRMA/EFPIA member and non-member trials.


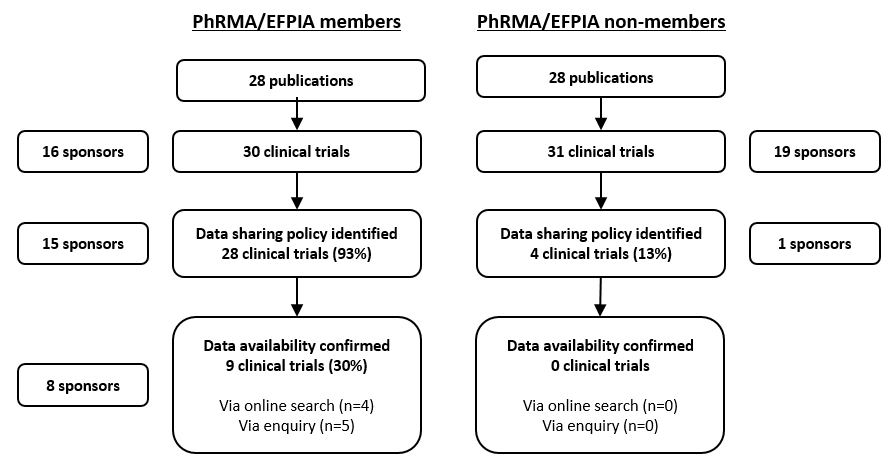


Table S2: Breakdown of PhRMA/EFPIA member and non-member clinical trials for which eligibility for data sharing was not confirmed

| **Reason** | **PhRMA/EFPIA member trials (n=30)** | **PhRMA/EFPIA non-member trials (n=31)** |
| --- | --- | --- |
| Confirmed data sharing not available | 11 (37%) | 15 (48%) |
| - Sponsor does not share IPD | 2 (7%) | 13 (42%) |
| - Study is still ongoing | 5 (17%) | 0 |
| - Medicine not approved by both EMA and FDA, or on-going regulatory submission | 4 (13%) | 1 (3%) |
| - Medicine no longer in development | 0 | 1 (3%) |
| Unable to confirm eligibility/ineligibility | 10 (33%) | 16 (52%) |
| - No response within 3 months to specified data sharing enquiry process^1,2^ | 6 (20%) | 4 (13%) |
| - No response within 3 months to generic sponsor contact^3^ | 0 | 12 (39%) |
| - Full research proposal required to assess eligibility | 4 (13%) | 0 |
| Data specified as: number of trials (% of the total number of trials assessed by PHRMA/EFPIA member status)  ^1^One clinical trial (conducted by a PhRMA/EFPIA non-member) was confirmed as ineligible for data sharing after the 3 month enquiry period had expired (confirmed 5 months after initial enquiry), on the basis that the trial was still ongoing.  ^2^Two clinical trials (one conducted by a PhRMA/EFPIA member, and one a PhRMA/EFPIA non-member) were confirmed eligible for data sharing after the 3 month enquiry period had expired (confirmed 5.3 and 6.6 months after initial enquiry)  ^2^No specific data sharing enquiry contact/process identified. | | |

Table S3: Breakdown of clinical trial completion statuses

| **Therapeutic area** | **Sampled** | **Trial ongoing at publication^1^** | **Trial ongoing 2 years after publication^1^** |
| --- | --- | --- | --- |
| Cardiovascular disease | 7 | 0 | 0 |
| Dermatology | 3 | 0 | 0 |
| Endocrine | 7 | 1 (14%) | 1 (14%) |
| Haematology | 7 | 3 (43%) | 2 (29%) |
| Infectious disease | 14 | 4 (29%) | 0 |
| Neurology | 1 | 0 | 0 |
| Oncology | 13 | 11 (85%) | 8 (62%) |
| Ophthalmology | 2 | 0 | 0 |
| Rheumatology | 7 | 2 (29%) | 2 (29%) |
| **Total** | **61** | **21 (34%)** | **13 (21%)** |
| Data specified as: number of trials by therapeutic area (% of the number of trials assessed by therapeutic area)  ^1^Trial ongoing as defined by completion date at ClinicalTrials.gov on 9 Aug 2017 | | | |

# References

1. **Clarivate Analytics. InCites Journal Citation Reports** [ h<ttps://jcr.incites.thomsonreuters.com/JCRJournalHomeAction.action?pg=JRNLHOME&year=2016&edition=SCIE&categories=PY#]>

2. **PMLiVE: Top 25 pharma companies by global sales** [ <http://www.pmlive.com/top_pharma_list/global_revenues>]
